# Pathologising ‘Refusal’: Prison, Health and Conscientious Objectors during the First World War

**DOI:** 10.1093/shm/hkac015

**Published:** 2022-03-24

**Authors:** Max Hodgson

**Keywords:** conscientious objectors, prisons, healthcare, pathology, First World War

## Abstract

This article examines the extent to which the refusals of British conscientious objectors (COs) to fight during the First World War were pathologised through the lens of physical and mental health, and the ways in which such a pathology impacted their treatment in penal establishments. It argues that the government compromised the physical *as well as* the mental health of absolutist COs. The article also analyses the effects of the state’s pathologising efforts upon objectors, and the methods through which the physical bodies of COs were utilised against, or annexed by, the authorities. Drawing on Cabinet Minutes, Prison Commissioner Reports, and COs’ personal letters and memoir materials, it suggests that the case of COs offers an interesting comparison with the complex ways in which the phenomenon of ‘shell-shock’ was beginning to be understood in both somatic and psychological terms.

Who sees them DIE?

   ‘Not I,’ says the Nation,

   *‘A pure fabrication!*

   *They’ve lost weight, we know—*

   *A few stones, or so,—*

   *And some have gone mad*

   *With the tortures they’ve had—*

   *But if some have died*

   *Such cases we hide—*

*And no one, you’ll notice, for Murder is tried!’* ^1^

       *The prisoner does not consult the doctor, the State pays the doctor and consults him about the prisoner*^2^

Almost 2 years after the outbreak of the First World War, Secretary of State for War David Lloyd George expressed the British government’s hostility towards conscientious objectors (COs). In July 1916, in light of the extraordinary casualty rates at the Somme,^3^ the ‘skrimshanking’ of COs, he claimed, was unacceptable, and he would

only consider the best means of making the path of that class a very hard one. I am not sure that sending them to the Army is the best way of doing it. There are other ways of doing it, and that is all I say now.^4^

A majority of COs, having been court-martialled and granted conditional exemption from armed service, accepted some form of alternative wartime work. However, a minority of around 1,500 were unwilling to compromise, and refused to aid the war effort in any capacity.^5^ It was this group of ‘absolutists’—formed principally by members of the leading anti-conscription organisation, the No-Conscription Fellowship (NCF), and the Fellowship of Reconciliation, whose ranks were drawn largely from the Independent Labour Party (ILP) and the Religious Society of Friends^6^—at whom Lloyd George’s anger was directed.

Since January 1916, under the Military Service Act, exemption from conscripted armed service could be sanctioned on grounds of ill health, economic hardship, essential civilian occupation and conscientious objection. Those who refused to engage with the exemption process, or who dismissed alternative service, were considered by many politicians to have both spurned a generous offer and demonstrated the insincerity of their convictions. In light of the sacrifices being made by British soldiers and civilians—whether in blood, declining living standards, suppressed wages, rising food prices or rationing—the campaign against those who appeared to be preventing victory was invigorated by Lloyd George’s comments. Absolutists who refused to obey army orders were often sentenced to long periods of confinement with hard labour in military detention barracks, sparking allegations of abuse and mistreatment.^7^

By the summer, the government opted to transfer absolutists to civilian control; the uncompromising resistance of the absolutists and the ‘inflexibility of military discipline’ pushed the Prime Minister, Herbert Asquith, to place COs under the responsibility of the Home Office. Seeking to avoid the use of the death penalty at the front, but wary of encouraging spurious claims to conscientious objection, COs sentenced by court martial were to have their cases reviewed by the Central Tribunal. Those considered genuine were to be released to work under a new Home Office Scheme, while those who were refused this classification returned to civil prison to complete their sentences.^8^ A high proportion of cases were recommended for release, but a substantial number of absolutists refused to compromise. They remained in prison, subjected to Lloyd George’s ‘hard path’.

Typically sentenced to 112 days’ imprisonment with hard labour alongside common criminals in the Third Division, absolutists served their sentences before being released back to the army for court-martialling again and re-sentencing. Hard labour no longer included heavy manual toil, such as the treadmill or the crank, but the first 28 days of a sentence were spent in isolation, the silence rule was imposed throughout, and general penal conditions were administered. Diet was basic, exercise restricted and plank beds without mattresses were the rule for the first fortnight.^9^ Absolutists resisted to different degrees: while many served their time with little obstruction, some refused to work, others refused to eat and many challenged the disciplinary regime. Punishment diet was administered for offenders, with forcible feeding for hunger strikers. From 1916, some absolutists spent close to 3 years in prison, and repeated sentences took their toll on their physical and mental health. Over 5,000 objectors were imprisoned throughout the war; hundreds were eventually released on the grounds of ill health and over 70 died, 9 within prison.^10^

The NCF was particularly vociferous in exposing the treatment of COs. Its leading lights, including its Chairman, Clifford Allen, a socialist internationalist with excellent contacts in the British labour movement; its Secretary, Fenner Brockway, editor of the ILP’s *Labour Leader*; and Stephen Hobhouse, a well-connected Quaker, pacifist and social worker, were extremely effective at highlighting the injustices and inconsistencies of the tribunal process. Despite this, both the Asquith and Lloyd George governments continually resisted any relaxation of prison discipline.^11^ In 1917, against the backdrop of an orchestrated campaign to highlight the effects of prison conditions on COs’ health (headed by Margaret Hobhouse, whose son Stephen had by this time been imprisoned for over a year), a meeting of the War Cabinet asserted that public opinion had hardened since the introduction of conscription, and COs were not to be relieved. According to Walter Long, who as President of the Local Government Board oversaw the operation of the Military Service Tribunals, ‘[E]ven if one of these Conscientious Objectors died in prison’, he ‘did not believe that there would be any substantial outcry’.^12^ The Cabinet considered that, in light of the ‘equality of sacrifice’ of British civilians, public opinion would be ‘very impatient with the lenient treatment’ accorded to COs, and actively agitated for propaganda against the ‘unpatriotic and dangerous’ men who made up the pacifist movement.^13^

Although many COs were treated with dignity and respect throughout the exemption process, others, gaining the attention of the press and liberal opinion, were ridiculed by military tribunals, dismissed as shirkers, cowards and ‘cultural criminals’.^14^ Historical accounts of COs have often focused on the personal and collective experiences of objectors, and have examined their reception by the public, their time in prison, and the significant impact they had on the post-war campaign for penal reform in Britain.^15^ In other studies, state motives have been scrutinised.^16^ As Cyril Pearce has noted, within accounts of the broader themes of war and peace, pacifism and conscience, the ‘finer detail is often obscured’.^17^ Commonly stereotyped as ‘weak-willed’ and mentally pusillanimous, the health of COs is a clear example of this obscuration: over a century on from the conflict little is known about the ways in which the experiences of COs in prison affected their physical and mental well-being, or the extent to which the British wartime state engaged with objectors’ health.

Drawing on a range of Cabinet minutes, Prison Commission reports, medical journals and the letters and memoirs of COs, this article examines the impact of prison conditions on the health of COs, and analyses the ways in which this was understood, engaged with and compromised by the state. It asks whether the health of COs was being deliberately compromised, and suggests that for a range of political, military, class and gender-based reasons, it became convenient for the government to pathologise the ‘refusals’ of COs to serve as *both* physical and mental weakness. First it considers the state’s rationale for pathologising refusal as physical as well as mental weakness. Next it asks how this pathology was operationalised, and reflects upon the state’s attempts to reconcile the competing objectives of exhibiting and punishing the ‘weakness’ of COs, with ameliorating their ‘softness’ through healthcare provision and rehabilitation. Finally, it examines the effects of the state’s pathologising efforts upon those who were imprisoned. While the article is concerned primarily with the prison experiences of absolutist COs, examples are also drawn from military tribunals and Home Office camps as broader indices of pathologisation.

The case of COs and the problems they posed for prison medical officers and the state reinforces the notion that medical beliefs are always underpinned by cultural attitudes and political values. As such, medicalised views of deviance expressed as ‘culturally and historically specific designations’ can have significant ramifications for the health of prison populations.^18^ This article demonstrates that the physical and mental health of absolutist COs was undermined by the government’s approach to them during the war. At times callous in its treatment, the government attempted to portray COs’ poor health as proof of their weakness. The temperaments, however, of tribunal panels, the conditions of carceral institutions and the efficacy of prison medical services differed, sometimes severely, across Britain; and this uneven playing field presents difficulties when trying to examine objectors’ agency in relation to the state’s pathologising efforts.^19^ The mixed class backgrounds of COs also presented a problem for the British state, and offer an interesting comparison with the evolving understandings, in both somatic and psychological terms, of the phenomenon of ‘shell-shock’, amid complicated changes in medical understandings of physical and mental health, illness and pathology.^20^

## Fin-de-Siècle Prison Health, Degeneration and Military Recruits

The physical health issues experienced by COs have not been examined in connection with historical studies of medicine and health in prisons. Indeed, while analyses of the historical management of the mental health of prisoners have proliferated in recent years, a thoroughgoing examination of physical health has been omitted.^21^ More broadly, scholars have identified an important dynamic in the historical interplay of prisoner health and healthcare provision: the conflicting priorities of the prison medical service.^22^ Throughout the nineteenth century, and particularly following the 1865 and 1877 Prison Acts, the authority of prison medical officers increased significantly.^23^ Prison doctors had the power to pass inmates unfit for labour, send them to hospital and to issue additional items of diet. In extreme cases they could recommend prisoners’ release on medical grounds.^24^ Following the Gladstone Committee of 1895, the power of the medical service was extended further, while at the same time ‘reform and discipline were collapsed to mean the same thing’.^25^ As a result, the prison medical service was effectively tasked with ‘refereeing the punitive excesses of those who administered the penal system’, and forced to navigate conflicting roles as disciplinary officers and healthcare providers within institutions ‘intentionally organised for the purpose of inflicting deterrent punishment’.^26^ While some historians have argued for the pre-eminence of the provision of health care in the work of prison medical officers, others contend that medical service personnel were powerful actors in the maintenance of the punitive regime.^27^ Moreover, as the image of penal establishments gradually moved ‘closer to that of the Health Service hospitals in Britain’, questions remained over the inclusion of ‘punishment’ within prisoner ‘treatment’, and thus over the role, significance and utilisation of medical care within prisons.^28^

These conflicting priorities were still evident at the outbreak of the Great War, but it was the longstanding concerns about the physical fitness and degeneration of the British state, within which war resistors became engulfed, that ensured they were brought to the fore by the problem of the CO. Between 1899 and 1902, the military set-backs of the Second Boer War intensified anxieties about the physical make-up of the British population. Suggestions that almost half the working population was unfit for military service intensified a debate that continued until the outbreak of the First World War.^29^ In the intervening period, serious questions were asked as to where the human material of the military would be sourced; and by the time fighting erupted in 1914, despite a rush of voluntary enlistment, close to half the men eligible to sign up had avoided doing so.^30^ Fear of death and injury, the potential for economic gain as a result of labour shortages at home and familial obligations all contributed to the enlistment troubles.^31^ In addition, just as the shortage of men at the turn of the century was blamed on the ‘inability of the working class to meet even the drastically reduced physical requirements’ necessitated by the war, so the authorities continued after the introduction of conscription in 1916 to lament the ‘existing “low category” of physical fitness for Military Service’.^32^

Concern about physical deficiency was not restricted solely to military contexts. A Royal Commission set up in 1903 to investigate the war in South Africa promptly connected the physical disabilities of ordinary British soldiers with working class conditions and issues of heredity.^33^ Recognising the increasing concerns about degeneration, *The Lancet* countered neo-Malthusian propositions to check the excessive fertility of the labouring poor by striking eugenic tones that encouraged the ‘fittest’ classes to ‘bestow more of their rejuvenating progeny on the flagging race’.^34^ The journal sought the configuration of ‘appropriate’ material for well-arranged marriages—‘to secure and keep up a class capable of government and legislation’—for, as the *British Medical Journal* also fretted, ‘if these [working class] men are unfit for military service, what are they good for?’^35^ As Daniel Pick notes, anxieties about degeneration reached their apotheosis with the Boer War, and the revival of the ‘Condition of England’ question was now ‘centrally concerned with the condition of the English body’.^36^ For the governing class, the ‘most damaging effects of the social problem were registered in the quality of the nation, the fitness of the race and the efficiency of the Empire’. Degeneration became ‘a medium by which respectable classes could articulate their hostility for culturally subversive elements of society’, and the threat of the feeble-minded and physically deficient was rolled into one within discussions of the identification and treatment of the problem.^37^ The rise of eugenics at the turn of the century encouraged the view that nervous disorders such as hysteria and neurasthenia signalled the beginning of biological, and therefore social, political, and imperial decline.^38^ These fears were compounded during the First World War, when only 59 per cent of British recruits were found to be of the requisite fitness to serve at home and abroad.^39^

These anxieties played out against a background of changing understandings of health in Britain, and stimulated new approaches to measuring the condition of the nation.^40^ A gradual turn towards state intervention occurred during a period of changing medical ideas, particularly in relation to psychosomatic illness, which was only further complicated by events of the First World War, the experience of diagnosing and treating ‘shell-shock’, and the increasing discussion of Freudian theories of psychoanalysis.^41^ Orthodox views of neuroses, psychoses, neurasthenia and hypochondriasis presumed functional, rather than psychological, foundations up until the mid-nineteenth century, but deliberations around both theories were amplified through the war.^42^ Furthermore, the proliferation of psychological interpretations offered opportunities for marking out ‘abnormalities’ within a populace—something that appealed to a British state concerned about managing its population.^43^ Where the authorities worried that the empire’s defence rested on men who lacked determination and nerve, they sought instead the physical vigour of the ‘Regency sportsman’ and military hero.^44^ A swathe of investigations were established in the ‘quest for national efficiency’; each battled with the issue of degeneration, and worries about the poor health of the population became entrenched.^45^ Richard Soloway argues that debates over deterioration in Britain were more to do with ‘anxieties about economic, social, political and cultural change’ than ‘quantifiable reality’.^46^ The conception of politico-social bodies as artefacts upon which to operate was indeed new in the nineteenth century, but across Europe the ‘traditional imagery of a metaphoric relation between the individual body and the social body’ was shifting towards ‘an argument that there existed an actual correlation between the two’.^47^ The creation of ‘human archives’ assisted authorities in knowing and ordering their worlds; and the increasingly interventionist practices of modern states complemented the changing ideas, in Britain at least, about the legitimate frontiers of medical intervention into the social body.^48^

In 1915, the War Policy Committee reported that close to 250,000 men had broken down physically in the early months of the war, and fears that inferior men were defending the empire appeared to be being realised.^49^ As ‘neurasthenics’—‘[s]tunted, weedy’, of ‘notably poor physique’—filled the beds of military hospitals and fears of an enormous loss of manpower abounded, the government voiced the alarming realisation that ‘an A1 Empire’ could not be sustained with a ‘C3 population’.^50^ The new phenomenon of ‘shell-shock’ further confused this picture. ‘The uncertainties of pre-war understandings of nervous and mental disorders carried over into war time’ and, unable to accurately understand the psychological dimensions of soldiers’ breakdowns, ‘the limitations of pre-war knowledge of hysteria and neurasthenia shaped early approaches to “shell-shock”’.^51^ Early analyses, proposed by physicians and neurologists like Dr Charles Myers and Sir Frederick Mott, focused on physical symptoms and commotional causes, with a particular emphasis upon the effects of artillery shell blasts in damaging men’s nerves. As Jessica Meyer argues, this physical explanation was used ‘to create an idea of a medical illness as the root cause for the rash unmilitary behaviour being witnessed by 1915’.^52^ By 1916, however, Myers had renounced his ‘crude neurological explanation’, and a year later was focused instead on the psychological underpinnings of the symptoms among British troops.^53^ Concerns about Britain’s health, then, were being framed—by different groups and at different times—in *both* physical and mental terms; workers and soldiers believed that ‘their desire to serve, their moral imperative, overwhelmed their physical shortcomings’, whereas medical discourse prior to 1914 had often implied that ‘neurotic Britons were not just ill or bad, but unpatriotic’. Despite the developments in medical discussions around shell-shock, those who were (or felt themselves) unable to serve the nation could still be cast as ‘enemy aliens’ at a biological level, to the extent that, as David Silbey notes, the government ‘quickly came to confuse physical inability with what they saw as an unwillingness to serve’.^54^ But where so many of the ‘unemployables’, who in earlier years bore the brunt of upper-class biological concern, had disappeared into the ranks of the army following conscription, those who actively resisted armed service were now made most visible.^55^

Worried about the pacifistic influence of COs, the sacrifices being made by the British population, and seeking to reinforce the image of a robust, vigorous Britain in the theatre of war, the government tried to frame COs’ unwillingness to serve as a symptom of both physical *and* mental weakness. By examining their prison experiences, their articulation as ‘physical … non-entities’, ‘effeminate, anaemic’ men, we can see this pathologisation far more clearly.

## Pathologising Refusal as Physical *and* Mental Weakness

Following the passing of the Military Service Act in January 1916, COs who were willing to engage with the exemption process were challenged to ‘prove’ their conscientious objection before military service tribunals. Tribunals were faced with hundreds of thousands of applications for exemption, a great number of which were granted to those in ill-health or working in essential occupations. With recent estimates of the numbers of COs reaching 20,000, the numbers applying for exemption on grounds of conscience formed only a small part of these appeals.^56^ As Cyril Pearce and Helen Durham have noted, though, there remains great difficulty in making accurate calculations; not all objectors appeared before tribunals, those who did often applied for exemption on multiple grounds, and questions remain as to how many COs remained ‘hidden’ within war-essential occupations.^57^ To complicate matters further, COs’ experiences before tribunals were mixed, influenced by class, political and geographical prejudices, as well as the changing exigencies of wartime.

As John Rae has demonstrated, while working under intense pressure tribunals granted some form of conditional exemption to over 80 per cent of COs who were subjected to their decisions.^58^ Yet objectors could also face great difficulties. The government’s definition of conscientious objection was clearly broader than the military would accept, and tribunal members were unafraid to demonstrate contempt for the inclusion of a conscience clause within the Military Service Acts.^59^*The Tribunal*, the organ of the NCF, reported regularly on the ‘scandalous maladministration of the Act’ and the propensity for tribunals to bully, interrogate and humiliate objectors.^60^ As Lois Bibbings has argued, tribunals became arenas for articulating the deviance of COs, and, despite the legal grounding of objection, these men were treated and represented as ‘cultural deviants’. Where newspapers fuelled British patriotism with stories of heroic soldiers at the front, objectors were interpreted through the language of degeneration.^61^ ‘[S]hirking, lazy, spineless, un-Christian, unpatriotic … womanly … or suspected of sexual inversion’, objectors were rendered to the British public via examinations of their ‘manhood’, with the implication that these were ‘not real men’, but ‘unmen’.^62^

The stereotyping of COs as mentally weak is a well-known component in this construction of deviance; but the physical health of objectors played a significant role too. Many objectors, for instance, sought exemption at tribunals on the grounds of *both* conscience and ill-health, and were subjected to a separate medical examination to determine their fitness to serve. Rowland Barrett, a Labour Party socialist and commercial traveller who appeared before Sunderland Tribunal in March 1916, arrived with a note signed by his civilian doctor certifying that he suffered from a disease of the thyroid ‘paired together with an inguinal rupture’. According to his civilian doctor, he was ‘totally unfit to be taken into the Army, and if he is subject to … heavy exertion he will [be] a physical wreck’.^63^ Newspapers covering the tribunal reported that Barrett ‘had to take the greatest care’ and had been certified unfit by a number of doctors.^64^ The tribunal responded by pointing out that Barrett had been passed fit by an army doctor, and his case was quickly dismissed. Barrett’s situation was afterwards raised in the House of Commons, where further light was shed on his ex-ophthalmic goitre, abnormal pulse and defective eyesight, but the Under Secretary of State for War saw no grounds for an enquiry.^65^ The dismissal of Barrett’s poor health and conscientious objection no doubt provided much publicity for the socialist–internationalist politics that underlay his approach to military service.

Other objectors had their refusals framed in both physical and psychological terms, castigated at tribunals in 1916 as ‘feeble folk’, unhealthy minds in unwholesome bodies and shivering masses of ‘unwholesome fat’.^66^ Through 1917, tribunal members railed at the ‘most awful pack that ever walked the earth’, the ‘breed’ of men, the ‘rot’, who ‘ought to be hanged’.^67^ When Emmanuel Ribiero, an absolutist of socialist and Methodist convictions, who had already been imprisoned for a year, was court-martialled in March 1917, officers were forced to assemble in the ward of Lord Derby Hospital as a consequence of Ribiero’s ill-health. Hunger striking in protest at the introduction of compulsion, Ribiero had been forcibly fed and was too weak to stand. He was sentenced to two further years’ imprisonment with hard labour, and would be forcibly fed over 150 times before his release in 1918.^68^ Like the Suffragettes before him, Ribiero hunger-struck to attract public attention and sympathy for his cause; and in similar terms, his forcible feeding can be read not as an attempt to preserve life, but rather to punish.^69^ So often these cases were associated with cowardice and shirking, or even suggestions of insanity; yet mixed in with these allegations were clear judgements of physical infirmity. The language of the tribunals habitually simulated nineteenth-century vernacular of physiological degeneration—‘want of stamina’, ‘flabby heart’, ‘underdeveloped musculature’.^70^ As Bibbings points out, however, COs were often caught within the bind of the state: where the absolutist refused to be forced into any work that assisted the military effort, he also refused state-sanctioned ‘solutions to degeneracy … suggested by those advocating (re)masculinisation’, and actually provided ‘support for his construction as in some [physical] way lacking as a man’.^71^

This blending of physical and psychological understandings of refusal could also be seen outside the tribunals. Medical journals often employed both physiological and psychological frameworks to associate neurasthenia with conscientious objection and weakness. Reporting on the ‘neurasthenia of the home forces’ in the summer of 1917, *The Lancet* noted that military doctors had met with few neurasthenics whose ‘physique or robustness was beyond the average. If the weight is occasionally excessive it is associated with flabbiness or want of tone’.^72^ In correspondence to the *BMJ*, a ‘Late Civil Surgeon’ struck similar tones when attempting to understand the potential for ‘emotional shock’ in inflicting neurasthenia. ‘[C]ongenital neurasthenia’ was, they suggested, a more accurate term ‘because of the strong hereditary element in the production of this lifelong condition’. There was no doubt in the surgeon’s mind that the war had brought these individuals into prominence, but they, ‘like the poor’, were always present in society.^73^ Often these neurasthenics could not be convinced that their ‘symptoms were *not* caused by disease of an organ’, but it was felt that:

Mentally, morally, *and* physically, these cases do not make good soldiers … and should be rejected at the beginning. They are, I suspect, the material out of which are made … most, of our conscientious objectors … so that their loss to the army would be practically nil.^74^

Environment and class also appeared to be important factors within discussions of both physiology and psychology. So often, it was deemed, neurasthenics, who ‘were sure of their unfitness for military service and did not conceal their resentment at being called up’, had not ‘benefited’ from a public school education:

All social classes are affected … But … the class who furnish the greatest number are they whose education has been carried … not yet far enough … The atmosphere of our public schools, in which character and manliness are developed side by side with learning, seems to prevent neurasthenia.^75^

These interpretations of neurasthenia among COs make for interesting comparison with historical understandings of neurosis within the armed forces. Early scholarship suggested that while symptoms of hysteria appeared primarily among enlisted soldiers, neurasthenic symptoms were more common among the officer class. As Elaine Showalter argued, this ‘extraordinarily tidy distribution of symptoms and diagnoses’ was ‘consistent with late Victorian moralistic and class-oriented attitudes to hysteria and neurasthenia’, such that neurasthenia among officers was often ‘interpreted as selfless and noble’.^76^ Even Dr W.H.R. Rivers, who developed Myers’ psychological theories and famously treated Siegfried Sassoon for shell-shock, stressed the frequency of the ‘hysterical’ private and the ‘neurasthenic’ officer.^77^ More recent research, however, has questioned this binary opposition, emphasising the diversity of servicemen affected by shell-shock, the far more nuanced diagnostic distinction between neurasthenic officers and hysterical soldiers, and the subtleties of contemporary medical practice.^78^ Meyer suggests that hysteria was, in any case, used as a diagnostic term ‘comparatively infrequently’. As knowledge of the psychological foundations of shell-shock developed, distinctions between hysteria and neurasthenia, the officer and enlisted soldier, the physical and the mental, became blurred, and new complexities regarding diagnosis and treatment arose around the axis of the un/conscious loss of self-control.^79^ Class-based interpretations proved equally problematic in the case of COs, some of whom hailed from the social strata which might usually be expected to form the officer corps. If the government, concerned about the fitness of the British state, wished to maintain an image of the strength of the ‘respectable classes’, publicly educated objectors could not be seen to demonstrate the existence of a ‘stigmatising feminine label of hysteria’ among upper-class, governing men. Neither, though, in line with contemporary accounts of neurasthenia, could they be articulated, like an ‘overworked neurasthenic officer’, as an ‘acceptable, even heroic male ideal’.^80^

Such understandings of physically ‘defective’ COs, then, relied in part on somatic considerations, but were complicated by psychological interpretations of health, heredity and environment, and concerns about class and degeneration. Timing was important too. Through 1917, following rapid increases in the numbers of psychological casualties returning to Britain, ‘softer’ curative regimes for the shell-shocked were pitched, including outdoor work and recreation, and therapeutic treatments.^81^ These ideas, in tension with the Army’s desire to return servicemen to the frontlines as quickly as possible, spilled over into debates on exemptions, and can be linked more broadly with a general feeling of war fatigue, a tempering of attitudes towards COs, and the release of some of the most prominent objectors from prison.^82^

As the government appeared to emphasise the physical incapacity of incarcerated absolutist objectors, prison medical officers were forced to referee the competing priorities of punishment, care and the desires of those who administered the penal system. If one adds to this the tensions of war, and, as so many organs of the press did, allegations of an unwillingness to do one’s ‘duty’, a toxic construction of conscientious objection, based on physiological as well as psychological deficiency, can be observed. Authorities looked to pathologise both the physical and psychological weaknesses of COs so that it remained possible, in narrative terms, for these men to be physically hardened and re-masculinised. These unsympathetic readings of objectors were to impact profoundly on the treatment of absolutists as they were sentenced to incarceration.

## Prisons and CO Health/Care

As thousands of COs filled Home Office camps and British prisons at any one time between 1916 and 1919, the government and prison authorities faced competing priorities. In light of the pathologisation of their refusals to fight, objectors’ frailties had to be exhibited to the public and appropriately punished. These weaknesses, though, also had to be ameliorated through healthcare provision and rehabilitation. British prisons were far better suited to the objective of punishment. Indeed, in the late nineteenth century, penal establishments in Britain were coming under sustained attacks for their alleged role in the destruction of prisoner health.^83^ These attacks continued through the turn of the century, as prisoners consistently suffered from a wide range of ailments related to weight loss, digestion, dyspepsia, influenza, bronchitis and rheumatism, as well problems of the lungs, eyes and nervous system.

Where the health of inmates was tested only by incidence of sickness, rates of mortality and prisoner weight loss, the causes were considered, principally, to be diet and discipline. In relation to diet, prison medical officers navigated various nineteenth-century edicts that either specifically prohibited the use of diet as ‘an instrument of punishment’, sometimes necessitated that diet *must* be penal, and at other times stood firm in the determination of diet by sentence, rather than prisoner necessity.^84^ This compromised the role of the prison medical service, as emphasis on care was subsumed under disciplinary penalty and the intention to ‘break the prisoner down’, ‘which in turn was underpinned by the doctrine of less eligibility’. Ensuring prisoners ‘could not receive medical care that was equivalent to or better than the care’ received by those outside the walls of the penitentiary, prisons became ‘sites of intervention’ that blurred ‘the lines between … medicine and … the jurisdiction of other authoritative bodies’.^85^

The balance between punishment, discipline and health, then, was complex. Most prisons had one medical officer, appointed by the Home Secretary (generally on the recommendation of the Prison Commissioners). These officers were frequently the most powerful officials in prison, yet remained under the commissioners’ control, their reports and recommendations subject to the commission’s censorship.^86^ Medical officers sought to equalise discipline and care, such that the dietary system was ‘adjusted to punish the prisoner with hunger, and yet stop short of injuring his health’, but too often the imbalance was obvious, the effects were deleterious and directly contravened the recommendations of the Gladstone Committee.^87^ Where the Gladstone report ‘finally devoted some thought to the question of how far prison conditions might themselves be productive of … disease’, prison authorities worried over accusations that their regimes produced illness, and continued to defend the system of discipline.^88^

Largely unchanged by 1916, this was the regime that received absolutist COs. Following their degrading treatment at tribunals, and as the government adjudged public opinion to have hardened, ample room was provided for the government to demonstrate the physical weakness of objectors.^89^ As military crises arose and the strains of war were felt by British civilians, prison authorities could also be seen to focus on CO’s health, and at times to vent their frustration at COs’ incapacity. In the reports of the prison commissioners during the early years of the conflict, it was related that receptions to prison had been ‘confined to the physically and mentally weak’. ‘The general standard of physique is now much inferior to … normal times, while the percentage of strong able-bodied men is comparatively small’. There was little doubt ‘that the vast majority of cases would have been found to be physically unfit’.^90^ Where ex-inmates had answered the call to arms, their bravery was hailed. Regular prisoners, the commissioners reported, had the desire to ‘do their bit’; absolutists, on the other hand, were castigated for preventing the release of further prison officers to the army.^91^

By March 1917, *The Tribunal* estimated that ‘between three and four thousand’ objectors were ‘either in prison’ or ‘Home Office work-centres for refusing to join the Army’.^92^ Most absolutists served sentences of imprisonment with hard labour, legally considered to be ‘the most severe form of imprisonment’ and limited to a maximum of 20 months. As they often served successive sentences, though, large numbers suffered for far longer periods of time. Many soon felt the effects of the penal regime. J.H. Collins, who had already suffered ‘double pneumonia’, could barely stand a day of punishment, and felt ‘confident’ the rest of his term ‘would kill me’. Upon inspection the prison doctor marked him fit and he was ordered to scrub floors and clean windows, despite being unable to lift a pail of water.^93^ J.A. Skinner suffered terribly from hunger and cold during successive sentences at Wormwood Scrubs and Wandsworth. Locked away in isolation following collapse and undiagnosed surgical tuberculosis, he required multiple operations and extended rest upon his release.^94^ COs at Durham and Liverpool prisons also suffered greatly, with up to one in six in the prison hospital at Liverpool; while those populating the Home Office camps were often ‘physically run down’ following prison emaciation. While bigger prisons had, on average, higher incidences of hospital referrals, conditions and health outcomes could vary according to prison doctors and institutional conditions.^95^ In the view of their sympathisers, though, the ‘breaking’ of absolutists left them ‘ready to die’ upon their release, and anyone with an existing ‘physical or nervous weakness’ was sure to collapse. Ribiero eventually dropped to seven stone in weight, and others complained that persistent requests to see the prison doctor resulted in reports to the governor and yet further punishment. Supporters of Clifford Allen, perhaps the best-known absolutist, feared that his punishment diet of ‘bread-and-water would lead to certain death at no very distant period’. *The Tribunal* was quick to allege that his ‘punishment, although said to involve speedy death, was continued in defiance of prison regulations’.^96^ Some absolutists stopped making medical requests, and the decline in their health allowed for their characterisation as ‘physical and moral degenerates’.^97^

The degradation of absolutists’ health reinforced the government’s construction of their refusal as a symptom of weakness, and highlighted the uncomfortable balance between punishing and ‘treating’ absolutists. The prison commissioners, for their part, bemoaned the 3,730 COs admitted to prison through 1916 and 1917, and stated openly that ‘[d]ietary restriction is the principal instrument of punishment’ for absolutists. Prison feeding was ‘intended to sustain life at a minimal ebb’, and while it had a debilitating effect on all prisoners, the cases of COs were more widely publicised; as historians have noted, absolutists were often clear in their commitment to breaking disciplinary rules, and thus consistently incurred dietary punishments.^98^ Interestingly, commissioners reported that for particular objectors—those ‘of superior class and education’—‘the question of dealing out … adequate punishment … is often beset with great difficulty’, and dietary punishment was used by prison governors to combat COs’ ‘constant conflict’.^99^ Here the language of degeneration, so often centred on anxieties about the biological inferiority of the labouring poor, was inverted to indict ‘upper-class’ COs, while falling short of ‘attributing degeneration to traditional [upper-class] morality’ in general.^100^ Indeed, in some cases, COs presented a problem: a number of absolutists *had* experienced what *The Lancet* hailed as the ‘atmosphere of our public schools, in which character and manliness are developed’, and yet still they refused to serve. In contrast, the prison commissioners hailed the way in which the war had demonstrated ‘the magnificent material of which the working-class of this country is composed’.^101^ Though absolutists hailed from all classes, a number (of often high-profile objectors) were neither working class nor, in the view of authorities, ‘magnificent material’, but products of the middle- and upper-class British state.^102^ They were not ‘selfless and noble’ neurasthenics, but were, in social terms, often too proximate to the ruling classes to be stigmatised with hysteria, and it thus became expedient to pathologise their refusals to fight as physical weakness. The reports of prison commissioners fulfilled this function, as the ‘inferior physique of the prison population’ was attributed to the increased proportion of incarcerated absolutists.^103^

In some instances of poor prisoner health—particularly cases of hunger striking—authorities released absolutists temporarily under the ‘Cat and Mouse Act’.^104^ Otherwise, the notion of ‘equality of sacrifice’ ensured there was little relief until the middle of June 1917. Where analysis of the appropriate treatment or punishment of shell-shock had developed to pivot on the issue of soldiers’ conscious or unconscious evasion of duty, discussions over COs tended to omit the possibility of ‘honourable’ status.^105^ Instead, absolutists voiced their fears and experiences of being hospitalised and forcibly fed, and the government, focused on their punishment and physical re-education, paid them little heed.^106^ Their approach was buttressed by the prison commissioners, who continued to claim that, despite net reductions in objectors’ diets, consistent weight loss among inmates of heights greater than 5′6″, and a higher ‘proportion of the daily average in hospital to the general prison population’ than any year on record, the health of COs was not being impacted. ‘[H]owever generous a diet’, they asserted, ‘certain prisoners would always lose weight’.^107^ Yet, as Graham noted, as many absolutists began their second year of imprisonment, ‘their weight when they entered on a new sentence was already reduced to its lowest safe minimum’. It was then ‘taken as their base … weight, and until they had lost considerably more they were not regarded as having lost weight and needing more food’.^108^ In the cases of hunger-striking absolutists across the country, many were forcibly fed, their supporters in parliament raising the spectre of prison doctors and authorities—who had ‘a very strong animus’ against COs—exercising ‘the powers of forcible feeding with the maximum amount of force and violence—almost cruelty’.^109^ The government’s compromising of COs’ health appeared to be reflected in the ease with which further reduced rations for prisoners were accepted and questions about the state of objectors consistently deferred.^110^

The government was forced to retain some caution, though. Through the efforts of the NCF—whose work as a lobbying and support organisation for incarcerated COs was so influential in pushing the government to transfer objectors to civil prisons and to establish the Home Office scheme—sustained reports of the ill-health of objectors and suggestions of the culpability of the state and prison authorities forced the government, after much hand-wringing, to mitigate prisoners’ circumstances. As war fatigue set in through 1917, the degradation of COs and their continued punishment began to impact public opinion. Protests grew, Margaret Hobhouse’s evocative account of the suffering of absolutists, *I Appeal Unto Caesar*, was published, and a number of concessions were granted to men who had served the equivalent of a 12-month sentence.^111^ Objectors serving second or subsequent sentences would no longer be required to spend their first month in solitary confinement; and in December 1917 Lord Curzon announced that, ‘from time to time’, the Home Office would report to the War Office those highlighted by prison medical officers for release on grounds of poor health.^112^ Simple measures of prisoners’ weights were taken, and, as *The Tribunal* put it:

Those whose health has been so broken in prison that the Government is afraid of the scandal that would result from their death may be liberated temporarily until such time as they have sufficiently recovered in health to be able to endure further torture.^113^

The Home Office promptly released Clifford Allen and Stephen Hobhouse, founders of the NCF, ‘not desiring, presumably, that two such well-known men should die in their hands’. This decision was much criticised, not least in relation to the privilege granted to objectors of ‘distinguished’ families.^114^ According to *The Tribunal*, ‘that done, they [the Home Office] relapsed into inactivity’.^115^

During the following 2 years objectors continued to suffer terribly in prisons, their health conditions ignored.^116^ The War Cabinet insisted in late 1917 that there would be no outcry if more objectors died in prison, and hard labour conditions were maintained; but between December 1917 and April 1918, 50 COs were released on the grounds of ill-health, and a total of 342 were released up to July 1919.^117^ The government still resisted where it could. In the spring of 1918, as the German army forced a major break in the Allied line, hostility towards COs increased, and the deaths of absolutists were played down. Before Arthur Horton died in Shrewsbury Prison in early 1918, he wrote that the prison regime was making him ‘as weak as a kitten’. Amid a cacophony of complaints against improper medical treatment, the inquest’s jury returned a verdict of death by natural causes.^118^ As the Armistice came and went and frontline demobilisation issues dominated, Cabinet members insisted that objectors had not been treated badly and should not be released. Even when military members of the Army Council acquiesced to objectors’ release, the Cabinet still refused.^119^ Under pressure, in September 1918 the government decided quietly—but unsuccessfully—to transfer remaining absolutists to the gaol at Wakefield, while across late 1918 and early 1919 a number of absolutists in Wandsworth Prison rebelled against their continued imprisonment and harsh discipline. Although the immediate effect was to increase the repression, the government was forced to pursue a more conciliatory line from the spring of 1919.^120^

Eventually, in mid-1919, the Cabinet agreed to release the remaining incarcerated objectors. The Home Secretary, Edward Shortt, remained suspicious of releasing COs on the grounds of ill-health, but the government worried increasingly that the resentencing of absolutists after the armistice would appear as if they were ‘resorting to persecution’.^121^ According to Hobhouse and Brockway’s post-war investigation, prison medical staff often proved inadequate, cursory in their treatment of the sick and under the thumb of the commissioners.^122^ The harsh treatment COs received was reflected in the prison statistics of 1920, where 11 regular prisoners were released on medical grounds, but 67 COs had to be discharged. The ‘disposal of COs’ was discussed and postponed frequently, before they were ‘discharged with ignominy … as incorrigible and worthless’.^123^ While much of the public may have seen in shell-shocked servicemen ‘cowards who demonstrated their lack of moral fibre’, COs pathologised by the state as physically *and* mentally weak were, it was alleged, proof that the ‘physical standard of the race’ still needed improving.^124^

## Internalising Pathologies

The physical effects of the state’s pathologisation of COs’ refusals, their treatment and care in prisons, are most evident in the cases of death and disease in carceral spaces, and the release of COs in poor health. The social and emotional impact of such physical pathologising, though, is harder to examine. As Joanna Bourke has stressed, historians should adopt ‘aesthesiological’ approaches to people in the past that acknowledge ‘the history of bodily and emotional reactions to the world’.^125^ In order to delegitimise conscientious objection, in cultural terms at least, authorities attempted to demonstrate the physical abnormality of the objector and to manage their physical health accordingly. ‘The prison became a laboratory in which the advice and expertise of the medical profession … was geared to reintegrating the confined back to normality’.^126^ But the state’s construction of ‘normality’ could be, and was, contested by COs, and the success of the state’s pathologising clearly depended, to a large degree, on the extent to which objectors themselves internalised this pathology.^127^ In what has elsewhere been termed a ‘hermeneutics of the soul’, prison conditions imposed upon inmates a self-examination designed to determine who was ‘worthy to belong to the brotherhood of the elect’: personal ‘trials of conscience’ and enquiries into ‘erring souls’ scrutinised objectors’ own understandings of their ‘duties toward the collective’, be that the state or the pacifist movement.^128^ The treatment of objectors in prisons was designed to induce introspection. The state sought to pressure COs to acknowledge their physical and mental weakness, to understand it as the foundation of their refusal to fight, and to accept the need for reconditioning and re-masculinising.

Previous studies have debated the effects of isolation and incarceration on the mental health of prisoners. Where scholars have demonstrated a clear association between prison conditions, separate confinement and mental deterioration, others have stressed the abilities of prisoners to resist, and even thrive, in isolation.^129^ Historical analysis of this kind attempts to find the ‘penal “truth”’ through understandings not of the authorities, but of ‘those who were interacting with’ the prison service. Obstacles arise, however, in attempting to identify, from memoir and autobiographical sources, genuine experiences of the ‘fortification’ of imprisoned COs, and reflections that have been cast, retrospectively, in the narrative of ‘robustness’ in order to disguise the invasive effects of the prison experience and state pathologisation. The tendency for COs to reflect frankly on their experiences suggests the validity of those who purport to have been strengthened through incarceration, and supports studies that attribute such resistance to a ‘strong sense of self’ and identification with a supportive group or organisation.^130^ Prison conditions, however, as well as prisoner agency, varied substantially across Britain.

For many COs, their prison experience and physical deterioration had severe effects. As their bodies broke down, they reflected on their identities, feelings of shame and degradation, and sometimes questioned their motivations.^131^ Isolation, poor diet, lack of exercise and mental stimulation saw objectors exhibit signs of memory loss, lethargy, dyspepsia and nervous collapse; many more were ‘broken in the fight for freedom’.^132^ Signs of the shame felt at their incarceration emerge in letters sent to family. ‘Dearly as I should love to see someone connected with my pre-prison existence’, wrote Barrett, ‘I don’t want anyone to come who would feel in any way tainted or disgraced by doing so’. Suffering from ‘a bad liver with enteritis’ and a hernia, he suggested to his correspondent that, ‘If you prefer it, I could probably have permission to write another letter in place of the visit’.^133^ Many lamented the indignity cast upon family members, felt their ‘manhood’ had been ‘degraded’, or were even driven to suicidal behaviour.^134^ Others internalised their weakness as pathological. ‘I can assure you’, wrote J.H. Collins, that ‘the life of a conscientious objector is almost unbearable’, a sentiment echoed by Alexander Neil Campbell, who feared that ‘he would be forced to give up the struggle … too weak to carry it on’.^135^

Some objectors demonstrated clear suspicions of medical interference by the state to control their bodies, while others related a ‘marked absence of sexual feelings, and a general diminution of virility’. As Hobhouse and Brockway noted in their post-war investigation, there was ‘widespread belief that drugs which are intended to act as sexual sedatives are secretly and indiscriminately administered in prison food’.^136^ Confidence, then, in the provision of medical care, and thus in the ability to resist the state’s pathologising, often appeared to depend on the individual medical officers. Physical ‘weakness’ appeared most evident where prison doctors were ‘surly, bad tempered, and forbidding’, while men ‘in good form’ attributed their resistance to ‘our largely excellent Doctor’.^137^ For those who suffered physiologically, the apparent disciplinary role of medical officers only exacerbated understandings of the state’s role in keeping objectors physically frail. Before Henry Firth died in 1918, he ‘became so ill and weak’ at Maidstone Prison, but was always treated as a malingerer. On account of such ‘unsympathetic treatment, he said he would not trouble to see the doctor again, as it was obvious that the latter did not intend to do anything for him’.^138^

Perhaps most significantly, though, as many objectors internalised accusations of weakness, their identities and convictions were undermined. Faced with long periods of self-examination and deteriorating physical health, Arthur Horton noted his loss of weight and vitality, while Hubert Peet acknowledged that the ‘prison regime provides every temptation to atrophy’. ‘Several times I felt acutely … that my pacifism might merely become passivism.’^139^ After just 1 day’s imprisonment and already feeling weak, J.H. Collins considered that, ‘[u]nder the current circumstances I think it most advisable to submit’.^140^ With greater foreboding, Paul Leo Gillian, a ‘delicate’ man ‘racked’ by prison, acknowledged that he emerged from Wormwood Scrubs ‘more dead than alive’; he died in Winchester Prison soon after.^141^ While the NCF attempted to monitor COs’ health, acknowledging their diminished physical capacity and establishing the ‘C.O. Convalescent Fund’, their efforts to prevent objectors internalising the state’s pathology were not always successful. Alexander Neil Campbell’s fear of being unable to carry on the struggle meant that he was in a ‘state of nervous collapse’ during his court martial. According to *The Tribunal*, in this ‘pitiable condition, he yielded to the persuasion of the Court, and consented to become a soldier’. Placed in the guardroom following his appearance, he committed suicide. *The Tribunal* related his death to the ‘long confinement and meagre diet’ having ‘done their work in reducing his physical powers’.^142^ For many objectors, then, the particular conditions imposed upon them rendered ‘health impossible’.^143^ Medical assistance appeared unavailable, or at least inferior to what could be expected outside of prison, and their poor state of physical well-being could erode conscientious convictions.^144^

For others, the pathologising interpretations of the state were less pervasive. It has been argued that, notwithstanding the harsh realities of the prison system, there were those who somehow found a way to cope, and some who even thrived within the isolation of the prison walls.^145^ In 1922 Hobhouse and Brockway noted that, despite the silence rule, dietary punishment and solitary confinement, many objectors found ways to ‘adapt’ to prison life and even achieved ‘feelings of personal accomplishment … and an improved internal life’.^146^ The ability to retain a sense of self, or to draw upon one’s identification with a group, was certainly a significant asset, and one that many COs, not least those of religious, pacifistic or socialistic persuasions, came to rely on. Indeed, objectors and their supporters often acknowledged state attempts to compromise their physical capacities, and in turn sought to obfuscate these efforts. In a message from Clifford Allen in 1916, the NCF was urged to do what it could to ‘preserve the physical fitness of the men in prison’, to prevent the authorities from rendering COs ‘useless for all forms of future service’.^147^ The association with a group identity could form a powerful well of resistance; by reinforcing the aims and convictions of objectors, COs were encouraged to accept their physical breakdown as a part of their sacrifice. As a result, the terms of the struggle were reoriented. No longer a battle centred upon ‘abnormal’, inferior, emasculated bodies, the pacifist’s ‘resistance is intellectual, moral, spiritual, not physical’.^148^ As Brockway defied attempts to break him through imprisonment in 1917, he reconstructed the parameters of the fight: ‘The Government may imprison my body, but it cannot imprison my mind and spirit’.^149^ Others deliberately adopted the language of disease and pathology to emphasise the strength of objectors. ‘[W]ith compulsion there arose a group of young men who took an option … morally and socially harder’, wrote an anonymous supporter.

But that disease of too much conscience, too much fellow-feeling, too nice an honour – it is a rare disease. It is so singularly lacking in infectious quality. So few of us catch it … It is not the danger which threatens nations.^150^

There of course remained concerns about objectors among their supporters. But others drew strength from their group identity, the expressions of sympathy, solidarity and encouragement providing inspiration and strength. In Winchester Prison a CO recognised that, ‘Although I’ve lost a lot of vitality, and a stone and a half in weight[,] I’ve not yet reached the physical collapse stage’. He was intent on combating the insufficient food, to ‘keep on and not bother the Home Office’, and urged the NCF to ‘concentrate your attention on the worst cases’.^151^ Civic organisations sent supportive messages to imprisoned COs, and often received replies that emphasised the inspiration objectors had taken from them. *The Tribunal* hailed the embarrassment that 1,500 men ‘“all out” against conscription’ were causing the authorities.^152^ Even as the silence rule prevented imprisoned objectors from communicating, they began to describe the feelings of unity in prison: ‘You cannot conceive the sense of spiritual exaltation and expansion received from the sight of those two hundred C.O.’s marching in step around the prison yard!’, wrote Brockway. ‘And when Sunday came … the joy of being one of the eight hundred C.O.’s there was almost intoxicating’. Clifford Allen ‘never gained so much inspiration than I have from these unconquerable men’, while others began to acknowledge their prisons as ‘spiritual universities’.^153^ In many cases, the longer men spent in prison, the more defiant they became. When Brockway was moved from Pentonville, Wandsworth and Wormwood Scrubs to Liverpool’s Walton Prison, he noted that he ‘had gone through my period of initiation’. He ‘no longer had the spiritual exultation of a novice … I would pit my wits against those of the authorities and defeat them whenever I could.’^154^

For those ‘reputation-mongers’ like Brockway and Allen, both socialists and founders of the NCF, their close-knit community, based upon ‘Socialist opinion and international faith’, ensured that their times in prison, while physically tough, provided fertile ground for agitation on issues of political, penal and social reform.^155^ Indeed, when Allen was first arrested, he wrote to colleagues from Maidstone Prison that he was ‘very glad to have been imprisoned with ordinary criminals’, since he was always ‘anxious … that the Socialists amongst the Conscientious Objectors should function very actively’.^156^ Undoubtedly, some posturing occurred among a number of CO accounts, whether focused on political agitation or, in the case of Hubert Peet, asserting his robustness through the claim that, ‘personally, I found I kept in the best health if I ate about two-thirds of my food’.^157^ Others, perhaps benefiting from some of the better conditions in certain British prisons, vowed ‘never [to] give in’, confident that they could ‘stand prison ad. lib. if necessary’.^158^ In spite of the issues of ‘less eligibility’, and the concern among COs that most had never met anyone ‘who was the better for being in prison’, a ‘fortunate few’ appeared to have been fortified by the experience. The ‘hard path’ upon which Lloyd George sought to place absolutists had clear and, for some, devastating effects upon their physical health. But a number of objectors strove resolutely to demonstrate their physical, moral and mental robustness.^159^

## Conclusion

Relative to the changing understandings of physical and psychological health in late-nineteenth and early twentieth-century Britain, the case of objectors presents an interesting comparison with evolving understandings of ‘shell-shock’ as a somatic and psychological phenomenon. Recent research that has teased out the nuanced medical understandings of, and approaches towards, these wartime conditions should now be utilised to improve our understandings of historical physical health in prisons.

The health of imprisoned COs was compromised during the First World War. The British government was anxious about the impact of COs on the morale of the nation, and responded by pathologising objectors’ unwillingness to serve as a *symptom* of physical and mental weakness. As a consequence of the state’s attempt to demonstrate absolutists’ ‘pathological weakness’, nine died in prison, and many more had to be released in severe ill health. The success of the government’s attempt to pathologise objectors’ health rested largely upon the extent to which COs internalised this pathology, and the memoirs, autobiographical materials and letters sent from prison provide a mixed bag of evidence. Prisoner agency never existed independently of carceral conditions, food provision or medical attention, each of which varied enormously across British institutions. As such, where objectors struggled against the intolerable pressure of the government and horrifying prison conditions, their bodies were co-opted by the state, determined for ‘reconditioning’ and ‘re-masculinisation’. Many took on forms of war work or re-enlisted; others suffered terribly and lost their lives. In contrast, some absolutists claimed to be fortified by their prison experience. By their own subjective accounts, they refused to be ‘reconditioned’. But by remaining at the mercy of a state-led prison medical service which underestimated their powers of resistance, their health was further compromised and paradoxically provided support for their construction as physically deficient.

More research on the physical health of prisoners is needed. The treatment of COs reinforces the notion that medical beliefs are always underpinned by cultural attitudes and political values, and demonstrates the different ways in which individuals have utilised their own bodies as sites of political and moral contestation, and have in turn had them annexed by the state. The state’s approach to absolutist COs fell uncomfortably between punishment and care, and their cases have significant implications for our understandings of the role of prison doctors as both care providers and disciplinary officers, and how the principle of ‘dual loyalty’ manifested itself historically.

## Footnotes

1 ‘CO’s in Prison, by the Mother of One of Them’, *The Tribunal*, 27 October 1917.

2 Mary Gordon, *Penal Discipline* (London: Routledge, 1922), 234.

3 On 1 July 1916, 19,000 British soldiers were killed at the Somme, with 57,000 injured. By November, the British had lost 420,000 men. See A. J. P. Taylor, *English History, 1914-1945* (Oxford: Clarendon Press, 1979), 60–61.

4 David Lloyd George, *War Memoirs*, 6 vols (London: Odhams Press, 1933), II, 712; HC Deb, 26 July 1916, vol. 84, c.1759.

5 The total number of COs is now estimated at around 20,000. See Cyril Pearce, *Communities of Resistance: Conscience and Dissent in Britain during the First World War* (London: Francis Boutle, 2020), 23; Cyril Pearce, *Comrades in Conscience: The Story of an English Community’s Opposition to the Great War* (London: Francis Boutle, 2001), 168–69, and the *Pearce Register of British First World War Conscientious Objectors*, hosted by the Imperial War Museum.

6 The Friends Service Committee, *The Absolutists’ Objection to Conscription:**A**Statement and an**Appeal to the**Conscience of the**Nation* (London: Direction of the Friends Service Committee, 1917).

7 John Rae, *Conscience and Politics* (London: Oxford University Press, 1970), 147–49.

8
*Ibid.*, 150, 154, 161–63.

9 Gilbert Thomas, *Autobiography, 1891-1941* (London: Chapman and Hall, 1946), 150–51.

10 Stephen Deakin, ‘Conscientious Objection to Military Service in Britain’, in Andrea Ellner, Paul Robinson and David Whetham, eds, *When Soldiers Say No: Selective Conscientious Objection in the Modern Military* (Surrey: Ashgate, 2014), 117; Rae, *Conscience*, 201; Fenner Brockway, *Inside the Left: Thirty Years of Platform, Press, Prison and Parliament* (London: George Allen & Unwin Ltd, 1942), 103; John W. Graham, *Conscription and Conscience: A History, 1916-1919* (London: George Allen & Unwin Ltd, 1922), 348.

11 Brace Committee, *The Home Office and Conscientious Objectors: A Report Prepared for the Committee of Imperial Defence. Part II, Conscientious Objectors in Prison* (London: Home Office, 1919), 1–2.

12 War Cabinet (hereafter WC) 257(3), 25 October 1917, 3–4, CAB 23/4, The National Archives, Kew. See also Margaret Hobhouse, *I Appeal Unto Caesar* (London: G. Allen & Unwin, 1917).

13 WC 142(14), 22 May 1917, 3, 5–7, CAB 23/2; WC 154(22), 5 June 1917, 6, CAB 23/3; WC 537(6), 26 February 1919, 5–6, CAB 23/9; WC 545(1), 17 March 1919, 1–2; WC 553(1), 3 April 1919, 1–2, CAB 23/10; WC 555(1), 10 April 1919, 1–3.

14 Lois Bibbings, ‘Images of Manliness: The Portrayal of Soldiers and Conscientious Objectors in the Great War’, *Social and Legal Studies*, 2003, 12, 343.

15 See The No-Conscription Fellowship, *The No-Conscription Fellowship: A Souvenir of**Its Work during the Years 1914-1919* (London: The Fellowship, 1919); Stephen Hobhouse and A. Fenner Brockway, eds, *English Prisons To-Day: Being the Report of the Prison System Enquiry Committee* (London: Longmans, Green & Co., 1922); Stephen Hobhouse, *The Silence System in British Prisons* (London: Reprinted by permission from The Friends’ Quarterly Examiner, 1918); Constance Braithwaite, *Conscientious Objection to Various Compulsions Under British Law* (York: William Sessions Limited, 1995); Lois S. Bibbings, *Telling Tales about Men: Conceptions of Conscientious Objectors to Military Service During the First World War* (Manchester: Manchester University Press, 2009); Lois S. Bibbings, ‘State Reaction to Conscientious Objection’, in Ian Loveland, ed, *The Frontiers of Criminality* (London: Sweet & Maxwell, 1995), 57–81; Thomas C. Kennedy, ‘Public Opinion and the Conscientious Objector, 1915–1919’, *Journal of British Studies*, 1973, 12, 105–19; Deakin, ‘Conscientious Objection to Military Service’; Will Ellsworth-Jones, *We Will Not Fight: The Untold Story of World War One’s Conscientious Objectors* (London: Aurum, 2008).

16 See Graham, *Conscription*, and David Boulton, *Objection Overruled: Conscription and Conscience in the First World War* (London: MacGibbon & Kee Ltd.,1967); Rae, *Conscience*; Keith Robbins, *The Abolition of War: The ‘Peace Movement’ in Britain, 1914-1919* (Cardiff: University of Wales Press, 1976); Brock Millman, *Managing Domestic Dissent in First World War Britain* (London: Frank Cass, 2000) and James McDermott, *British Military Service Tribunals, 1916-1918: ‘A**Very**Much**Abused**Body of**Men’* (Manchester: Manchester University Press, 2011).

17 Cyril Pearce, ‘Writing about Britain’s 1914-18 War Resisters’, *Reviews in History* (2015), 1-16, 2.

18 Peter Conrad and Joseph W. Schneider, *Deviance and Medicalization: From Badness to Sickness* (Philadelphia: Temple University Press, 1992), 17.

19 See Joanna Bourke, *Dismembering the Male: Men’s Bodies, Britain and the Great War* (Chicago: University of Chicago Press, 1996); William Murphy, *Political Imprisonment and the Irish, 1912-1921* (Oxford: Oxford University Press, 2014).

20 Tracey Loughran, *Shell-Shock and Medical Culture in First World War Britain* (Cambridge: Cambridge University Press, 2016), 77.

21 Catherine Cox and Hilary Marland, ‘Unfit for Reform or Punishment’: Mental Disorder and Discipline in Liverpool Borough Prison in the Late Nineteenth Century’, *Social History*, 2019, 44, 173–201; Catherine Cox and Hilary Marland, ‘Broken Minds and Beaten Bodies: Cultures of Harm and the Management of Mental Illness in Mid- to Late Nineteenth Century English and Irish Prisons’, *Social History of Medicine*, 2018, 31, 688–710; Catherine Cox and Hilary Marland, ‘“He Must Die or Go Mad in This Place”: Prisoners, Insanity, and the Pentonville Model Prison Experiment, 1842-52’, *Bulletin of the History of Medicine*, 2018, 92, 78–109; Stephen Watson, ‘Malingerers, the “Weakminded” Criminal and the “Moral Imbecile”: How the English Prison Medical Officer became an Expert in Mental Deficiency, 1880-1930’, in Michael Clark and Catherine Crawford, eds, *Legal Medicine in History* (Cambridge: Cambridge University Press, 1994), 223–41; Michael Ignatieff, *A Just Measure of Pain. The Penitentiary in the Industrial Revolution 1750–1850* (New York: Pantheon Books, 1978); Ian O’Donnell, *Prisoners, Solitude and Time* (Oxford: Oxford University Press, 2014); U. R. Q. Henriques, ‘The Rise and Decline of the Separate System of Prison Discipline’, *Past and Present*, 1972, 54, 61–93; Sean McConville, *English Local Prisons, 1860–1900: Next Only to Death* (London: Routledge, 1995).

22 Richard Smith, *Prison Health Care* (London: British Medical Association, 1984); Richard Creese, W. F. Bynum and J. Bearn, eds, *The Health of Prisoners: Historical Essays* (Amsterdam: Rodopi, 1995).

23 Martin Weiner, *Reconstructing the Criminal: Culture, Law, and Policy in England, 1830-1914* (Cambridge: Cambridge University Press, 1990), 309; Anne Hardy, ‘Development of the Prison Medical Service, 1774-1895’, in Creese, Bynum and Bearn, *Health of Prisoners*, 59–82.

24 Philip Priestley, *Victorian Prison Lives: English Prisons Biography, 1830-1914* (London: Pimlico, 1999), 169.

25 Peter Young, ‘Sociology, the State and Penal Relations’, in David Garland and Peter Young, eds, *The Power to Punish: Contemporary Penality and Social Analysis* (London: Heineman, 1983), 97.

26 John Gunn *et al.*, *Psychiatric Aspects of Imprisonment* (London: Academic Press, 1978), 5, 9; ‘Report from the Departmental Committee on Prisons’, *Parliamentary Papers*, vol. 19, 1895, 8; *The Lancet*, 14 December 1878, 859.

27 Hardy, ‘Development of the Prison Medical Service’, 59–82; Peter McRorie Higgins, *Punish or Treat? Medical Care in English Prisons, 1770-1850* (Oxford: Trafford Publishing, 2007), 236; Ignatieff, *Just Measure of Pain*, 59–62; Joe Sim, *Medical Power in Prisons: The Prison Medical Service in England, 1774-1989* (Milton Keynes: Open University Press, 1990).

28 W. J. Gray, ‘The English Prison Medical Service: Its Historical Background and More Recent Developments’, in G. E. Wolstenholme and Maeve O’Connor, eds, *Medical Care of Prisoners and Detainees* (Amsterdam: Associated Scientific Publishers, 1973), 132. See also Janet Weston, *Medicine, the Penal System and Sexual Crimes in England, 1919-1960s: Diagnosing Deviance* (London: Bloomsbury Academic, 2017).

29 See Arnold White, ‘The Cult of Infirmity’, *National Review*, 1899, 34, 239–41; Arnold White, *Efficiency and Empire* (London: Methuen & Co., 1901), 101–02; G. R. Searle, *The Quest for National Efficiency: A Study of British Politics and Political Thought, 1899-1914* (Berkeley: University of California Press, 1971), 35–53; Roderick Floud, *Height, Weight, and Body Mass of the British Population since 1820* (Cambridge, MA: National Bureau of Economic Research, 1998), 34–36.

30 John F. Maurice, ‘Where to Get Men?’, *Contemporary Review*, 1902, 81, 78–86; Braithwaite, *Conscientious Objection*, 126.

31 Adrian Gregory, *The Last Great War: British Society and the First World War* (Cambridge: Cambridge University Press, 2014), 89–95; Linda Maynard, *Brothers in the Great War: Siblings, Masculinity and Emotion* (Manchester: Manchester University Press, 2020).

32 Richard Soloway, ‘Counting the Degenerates: The Statistics of Race Deterioration in Edwardian England’, *Journal of Contemporary History*, 1982, 17, 142; WC 142(14), 22 May 1917, 3, 6, CAB 23/2.

33 Richard Price, *An Imperial War and the British Working Class: Working Class Attitudes and Reactions to the Boer War, 1899-1902* (London: Routledge & Kegan Paul, 1972), 134–35.

34 Richard A. Soloway, *Demography and Degeneration: Eugenics and the Declining Birthrate in Twentieth-Century Britain* (Chapel Hill: University of North Carolina Press, 1995), 40–41, 87.

35
*The Lancet*, 18 January, 1873, 102; *British Medical Journal* (hereafter *BMJ*), 15 July 1903, 202.

36 Daniel Pick, *Faces of Degeneration: A European Disorder, c.1848-1918* (Cambridge: Cambridge University Press, 1989), 195, 202.

37 David Garland, *Punishment and Welfare: A History of Penal Strategies* (Aldershot: Gower, 1985), 131, 223–24; Frederick H. White, *Degeneration, Decadence and Disease in the Russian Fin de Siècle: Neurasthenia in the Life and Work of Leonid Andreev* (Manchester: Manchester University Press, 2014), 30; *Lancet*, 17 October 1908, 1156.

38 Loughran, *Shell-Shock*, 74–75.

39 James J. Harris, ‘H1N1 in the “A1 Empire”: Pandemic Influenza, Military Medicine, and the British Transition from War to Peace, 1918-1920’, *Social History of Medicine*, 2018, 33, 618; J. M. Winter, ‘Military Fitness and Civilian Health in Britain during the First World War’, *Journal of Contemporary History*, 1980, 15, 211–44.

40 Daniel M. Fox, *Health Policies, Health Politics: The British and American Experience, 1911-1965* (Princeton, NJ: Princeton University Press, 1986), 15; Christopher Addison, *The Health of the People* (London: London University Press, 1914), 13; *BMJ*, 15 July 1903, 202; 14 February 1914, 379; *Lancet*, 14 April 1917, 579.

41
*BMJ*, 2 August 1919, 157. See Charles S. Myers, *Shell-Shock in France* (Cambridge: Cambridge University Press, 1940), 40; W. H. R. Rivers, *Instinct and the Unconscious*, 2nd edn (Cambridge: Cambridge University Press, 1922), 209–10, 218, 225.

42 Elaine Showalter, *The Female Malady: Women, Madness and English Culture, 1830-1980* (London: Virago, 2014), 167–70; Janet Oppenheim, *“Shattered Nerves”: Doctors, Patients, and Depression in Victorian England* (Oxford: Oxford University Press, 1991), 143.

43 Mathew Thomson, *Psychosocial Subjects: Identity, Culture, and Health in Twentieth-Century Britain* (Oxford: Oxford University Press, 2006), 6; Anna Davin, ‘Imperialism and Motherhood’, in R. Samuel, ed, *Patriotism: The Making and Unmaking of British National Identity*, 3 vols (London: Routledge, 1989), I, 204.

44 Oppenheim, *“Shattered Nerves”*, 146, 150–51.

45 See Soloway, ‘Counting the Degenerates’, 141–55.

46
*Ibid.*, 159.

47 Peter Holquist, ‘State Violence as Technique: The Logic of Violence in Soviet Totalitarianism’, in David Hoffman, ed, *Stalinism: The Essential Readings* (Melbourne: Blackwell Publishing, 2003), 135–36, 148.

48
*Ibid.*, 136, 142, 148–49; Sheila Fitzpatrick, ‘“Ascribing Class”: The Construction of Social Identity in Soviet Russia’, *Journal of Modern History*, 1993, 65, 745–70; Sim, *Medical Power*, 72.

49 R. J. Clare, ‘“Fit to Fight?” How the Physical Condition of the Conscripts Contributed to the Manpower Crisis of 1917-18’, *Journal of the Society for Army Historical Research*, 2016, 94, 232.

50
*Lancet*, 16 June 1917, 907; Harris, ‘H1N1 in the “A1 Empire”’, 618.

51 Loughran, *Shell-Shock*, 78.

52 Jessica Meyer, ‘Shell Shock as a Self-Inflicted Wound, 1915-1921’, in Laura Salisbury and Andrew Shail, eds, *Neurology and Modernity: A Cultural History of Nervous Systems, 1800-1950* (London: Palgrave, 2010), 233.

53 Meyer, ‘“Gladder To Be Going Out than Afraid”: Shellshock and Heroic Masculinity in Britain 1914-1919’, in Jenny Macleod and Pierre Purseigle, eds, *Uncovered Fields: Perspectives in First World War Studies* (Leiden: Brill, 2003), 198.

54 Loughran, *Shell-Shock*, 78; David Silbey, ‘Bodies and Cultures Collide: Enlistment, the Medical Exam, and the British Working Class, 1914-1916’, *Social History of Medicine*, 2004, 17, 63–64.

55 Gareth Stedman Jones, *Outcast London: A Study in the Relationship Between Classes in Victorian Society* (New York: Pantheon Books, [1971] 1984), 336.

56 Adrian Gregory, for instance, notes that in Huddersfield (‘a hotbed of pacifism’), ‘less than 1% of appeals to the tribunal were made on conscientious grounds’. Gregory, *The Last Great War*, 101.

57 See McDermott, *British Military Service Tribunals*; Pearce, *Communities of Resistance*; Cyril Pearce and Helen Durham, ‘Patterns of Dissent in Britain during the First world War’, *War and Society*, 2015, 34, 2, 140–59.

58 Rae, Conscience, 131–33.

59 Graham, *Conscription*, 111; Rae, *Conscience*, 72–73, 88; *The Tribunal*, 15 March 1916.

60
*Tribunal*, 8 March 1916.

61 Bibbings, *Telling Tales*, 52, 72, 92–93.

62
*Ibid.*, 103; Sandra M. Gilbert, ‘Soldier’s Heart: Literary Men, Literary Women, and the Great War’, *Signs*, 1983, 8, 423.

63 ‘Dr Alfred Salter: Certificate as to Barrett’s Ill Health’, 24 October 1916, Rowland Barrett Papers, MSS.83/3/PR/1, Modern Records Centre (MRC), University of Warwick (hereafter Barrett Papers).

64
*Sunderland Daily Echo*; *Newcastle Daily Chronicle*, 15 March 1916.

65 ‘House of Commons Questions on Barrett’s Individual Position’, 23–24 March 1916, Barrett Papers.

66
*Daily Sketch*, 10 April 1916.

67
*Tribunal*, 25 October 1917.

68
*Ibid.*, 20 June 1918; *Pearce Register*.

69 See J. F. Geddes, ‘Culpable Complicity: The Medical Profession and the Forcible Feeding of Suffragettes, 1909-1914’, *Women’s History Review*, 2008, 17, 1, 79–94. Forcible feeding involved the passing of a tube down into the stomach, through which food was delivered. Generally carried out twice a day, it was often performed by inexperienced prison medical officers on struggling patients.

70
*Lancet*, 1 June 1866, 691; ‘Court-Martial at Norton Barracks: Nine Conscientious Objectors’, 1917, Corder Catchpool Papers, Swarthmore College Peace Collection (SCPC), Pennsylvania (hereafter Catchpool Papers).

71 Bibbings, *Telling Tales*, 118.

72
*Lancet*, 16 June 1917, 907.

73
*BMJ*, 22 June 1918, 710.

74
*Lancet*, 16 June 1917, 907; *BMJ*, 22 June 1918, 710 (my emphases).

75
*Lancet*, 16 June 1917, 910.

76 Showalter, *Female Malady*, 174–75.

77 Meyer, ‘Shell Shock’, 234.

78 See Fiona Reid, *Broken Men: Shell Shock, Treatment and Recovery in Britain, 1914-1930* (London: Continuum, 2010); Hans-Georg Hofer *et al.*, *War, Trauma and Medicine in Germany and Central Europe, 1914-1939* (Freiburg: Centaurus, 2011); Paul Lerner, *Hysterical Men: War, Psychiatry, and the Politics of Trauma in Germany, 1890-1930* (Ithaca, NY: Cornell University Press, 2003); Fiona Reid and Christine Van Everbroeck, ‘Shell Shock and the Kloppe: War Neurosis amongst British and Belgian Troops during and after the First World War’, *Medicine, Conflict and Survival*, 2014, 30, 252–75; Stefanie C. Linden, Volker Hess and Edgar Jones, ‘The Neurological Manifestations of Trauma: Lessons from World War I’*, European Archives of Psychiatry an Clinical Neuroscience*, 2011, 263, 253–64; Stefanie Caroline Linden and Edgar Jones, ‘German Battle Casualties: The Treatment of Functional Somatic Disorders during World War I’, *Journal of the History of Medicine and Allied Sciences*, 2012, 64, 628–58.

79 Meyer, ‘Gladder’, 198–200; Meyer, ‘Shell Shock’, 237–42. Gundula Gahlen, ‘“Always Had a Pronouncedly Psychopathic Predisposition”: The Significance of Class and Rank in First World War German Psychiatric Discourse’, in Jason Crouthamel and Peter Leese, eds, *Psychological Trauma and the Legacies of the First World War* (Basingstoke: Palgrave MacMillan, 2016), 81–113.

80 Showalter, *Female Malady*, 174–75.

81 Treatments were predominantly physical, rather than psychological. See Stefanie Caroline Linden, ‘Triggers and Clinical Presentations of Functional Neurological Disorders: Lessons from World War 1’, *European Neurology*, 2020, 83, 174–81.

82 Peter Leese, *Shell Shock: Traumatic Neurosis and the British Soldiers of the First World War* (Basingstoke: Palgrave, 2002), 57–65.

83
*Lancet*, 8 February 1873, 221; 14 December 1878, 859; 17 January 1880, 104; 31 July 1880, 182.

84 Priestley, *Victorian Prison Lives*, 151, 155–56.

85 Joe Sim, ‘The Prison Medical Service and the Deviant, 1895-1948’, in Creese, Bynum and Bearn, *The Health of Prisoners*, 105; Cox and Marland, ‘Broken Minds’, 691.

86 Hobhouse and Brockway, *English Prisons To-Day*, 59–61.

87
*Lancet*, 10 June 1882, 968.

88 Gunn *et al.*, *Psychiatric Aspects*, 9; Priestley, *Victorian Prison Lives*, 180; Cox and Marland, ‘Broken Minds’, 696–97.

89 WC 257(3), 25 October 1917, 3–4, CAB 23/4.

90
*Report of the Commissioners of Prisons and the Directors of Convict Prisons, with Appendices (For the Year ended 31^st^ March 1916)* (hereafter *RCP*) (London: His Majesty’s Stationary Office, 1916), 6.

91
*RCP* (1915), 8–9; (1916), 6, 12–13, 20–21. See also Cameron McKay, ‘“Likely to Make Good Soldiers”: Mobilizing Britain’s Criminal Population during the First World War’, *Historical Research*, 2021, 94, 265, 578–600.

92
*Tribunal*, 8 March 1917.

93 ‘Letter from J.H. Collins’, 28 April 1916; 1 May 1916, Alexander and Edith J. Wilson Papers, SCPC (hereafter Wilson Papers).

94 Graham, *Conscription*, 278–79.

95
*Ibid.*, 145–56, 275; Pearce, *Communities*, 71, 356.

96
*Tribunal*, 12 July 1917.

97 Graham, *Conscription*, 277, 297; Pearce, *Communities*, 271, 420.

98
*RCP* (1917), 11; Pearce, *Communities*, 70; Graham, *Conscription*, 111; Rae, *Conscience*, 149.

99
*RCP* (1917), 11.

100 George Robb, ‘The Way of All Flesh: Degeneration, Eugenics, and the Gospel of Free Love’, *Journal of the History of Sexuality*, 1996, 6, 590.

101
*RCP* (1916), 13–14.

102 For instance, Stephen Hobhouse, Clifford Allen, Fenner Brockway and Hubert Peet. See the *Pearce Register*.

103
*Ibid.*, (1917), 5, 20; (1918), 14–15, 21; (1919), 12.

104 The Prisoners (Temporary Discharge for Ill-Health) Act of 1913.

105 Meyer, ‘Shell Shock’, 234–35.

106 Henry T. Hodgkin and Richard Roberts, ‘Some Typical Cases of COs’, 30 May 1916, 4; ‘Wilfred Hammon to His Mother’, n.d., Wilson Papers.

107
*RCP* (1917), 20–22; (1918), 22–24.

108 Graham, *Conscription*, 309–10.

109 HC Deb, 5 March 1919, vol. 113, cc.560–62.

110
*RCP* (1918), 22–23; WC 124(18), 23 April 1917, 6, CAB 23/2; WC 246(1), 8 October 1917, 2, CAB 23/4; WC 504(7), 19 November 1918, 5, CAB 23/8; WC 545(1), 17 March 1919, 2–3, CAB 23/9.

111 Graham, *Conscription*, 297–99.

112
*Tribunal*, 12 July 1917; HC Deb, 4 December 1917, vol. 27, c.56.

113
*Tribunal*, 13 December 1917.

114 Rae, *Conscience*, 225.

115
*Ibid.*, 27 December 1917.

116
*Ibid.*, 10 January 1918.

117 WC 257(3), 25 October 1917, 3–4, CAB 23/4; Graham, *Conscription*, 350.

118
*Tribunal*, 24 January 1918; 14 February 1918; 19 September 1918.

119
*RCP* (1916), 13; (1920), 17; WC 537(6), 26 February 1919, 6, CAB 23/9; WC 553(1), 3 April 1919, 2, CAB 23/10.

120 See Steve Illingworth, ‘The Rebellion of the “Basement Lecturers”: The Wandsworth Prison Disturbances of 1918-19’, *Prison Service Journal*, 2020, 249, 17–23.

121 HC Deb, 5 March 1919, vol. 113, c. 569.

122 Hobhouse and Brockway, *English Prisons To-Day*, 260–62, 610.

123 WC 555(1), 10 April 1919, 1–3, CAB 23/10; WC 537(6), 26 February 1919, 5; WC 504(7), 19 November 1918, 5, CAB 23/8; *RCP* (1920), 18.

124 Oppenheim, *“Shattered Nerves”*, 151–52; *RCP* (1919), 6.

125 Joanna Bourke, ‘How Do Physical Bodies Affect Cultural Change?’, in Harriet Swain, ed, *Big Questions in History* (London: Vintage, 2006), 238.

126 Sim, *Medical Power*, 9.

127 Dimitrios Theodossopoulos, ‘On De-Pathologizing Resistance’, *History and Anthropology*, 2014, 25, 419.

128 Igal Halfin, ‘Looking into the Oppositionists’ Souls: Inquisition Communist Style’, *Russian Review*, 2001, 60, 316–17; Igal Halfin, *Intimate Enemies: Demonizing the Bolshevik Opposition, 1918-1928* (Pittsburgh: University of Pittsburgh Press, 2007), 285.

129 Cox and Marland, ‘Broken Minds’; Marland, ‘“Close Confinement Tells Very Much upon a Man”: Prison Memoirs, Insanity, and the Late Nineteenth- and Early Twentieth-Century Prison’, *Journal of the History of Medicine and Allied Sciences*, 2019, 74, 267–91; O’Donnell, *Prisoners*.

130 O’Donnell, *Prisoners*, 60–84.

131 Hobhouse and Brockway, *English Prisons To-Day*, 641.

132
*Tribunal*, 26 July 1917.

133 ‘Barrett to M. Graham’, 8 May 1917, Barrett Papers.

134 Bibbings, *Telling Tales*, 68, 102.

135 ‘Letter from J.H. Collins’, 5 May 1916, Wilson Papers; *Tribunal*, 26 July 1917.

136 Hobhouse and Brockway, *English Prisons To-Day*, 586.

137 Hobhouse and Brockway, *English Prisons To-Day*, 638; ‘Claude to Frank’ January 1919, Wilson Papers.

138
*Tribunal*, 14 February 1918.

139
*Ibid.*, 24 January 1919, 3; Hubert W. Peet, *112 Days’ Hard Labour: Being**Some Reflections on the**First of**My Sentences as a Conscientious Objector* (London: Ploughshare, 1917), 14.

140 ‘Letter from J.H. Collins’, 28 April 1916, Wilson Papers.

141
*Tribunal*, 4 April 1918.

142
*Ibid.*, 23 January 1919; 1 May 1919; 26 July 1917.

143
*The Granite Echo*, I (October 1916).

144 Hobhouse and Brockway, *English Prisons To-Day*, 269.

145 O’Donnell, *Prisoners*, 36.

146 Hobhouse and Brockway, *English Prisons To-Day*, 581; Peter Brock, ‘Prison Samizdat of British Conscientious Objectors in the First World War’, *Journal of Prisoners on Prisons*, 2003, 12, 10.

147
*Tribunal*, 14 December 1916.

148
*Ibid.*, 14 August 1916.

149 ‘A. Fenner Brockway to Central Tribunal’, 17 January 1917, A. Fenner Brockway Collected Papers, SCPC.

150 ‘From the Trenches to Prison’, n.d., Catchpool Papers.

151
*Ibid.*, 24 January 1918.

152 ‘Sidcot Old Scholars Association to Corder Catchpool’, 1919, Catchpool Papers; *Tribunal*, 20 February 1919.

153 Edward W. Evans and Norman M. Thomas, eds, *Pioneers of Good Will: Statements of English Conscientious Objectors in Prison* (New York: Fellowship of Reconciliation, n.d.), 4.

154 Brockway, *Inside the Left*, 95.

155 Evans and Thomas, *Pioneers of Good Will*, 4; No-Conscription Fellowship newsletter, 31 January 1916, Library of the Religious Society of Friends in Britain, London (henceforth LRSFB); No-Conscription Fellowship, *The Position of the Conscientious Objector* (n.d.); Brockway, *Inside the Left*, 124–25.

156 ‘Letter from Clifford Allen’, 12 February 1917, TEMP MSS 977/1/7, Arnold Rowntree papers, LRSFB.

157 Peet, *112 Days*, 6.

158 ‘Claude to Frank’, January 1919, Wilson Papers.

159 Peet, *112* Days, 13.

## Notes

Dr Max Hodgson is a Research Fellow at the Centre for the History of Medicine, University of Warwick, working on the Wellcome Trust Investigator Award, ‘Prisoners, Medical Care and Entitlement to Health in England and Ireland, 1850–2000’. He has published on histories of penal reform in British and Soviet contexts, as well as the relationships between British and Bolshevik socialists.

